# Zfrp8/PDCD2 Interacts with RpS2 Connecting Ribosome Maturation and Gene-Specific Translation

**DOI:** 10.1371/journal.pone.0147631

**Published:** 2016-01-25

**Authors:** Svetlana Minakhina, Tatyana Naryshkina, Neha Changela, William Tan, Ruth Steward

**Affiliations:** Waksman Institute, Rutgers, The State University of New Jersey, Piscataway, New Jersey, United States of America; University of Toronto, CANADA

## Abstract

Zfrp8/PDCD2 is a highly conserved protein essential for stem cell maintenance in both flies and mammals. It is also required in fast proliferating cells such as cancer cells. Our previous studies suggested that Zfrp8 functions in the formation of mRNP (mRNA ribonucleoprotein) complexes and also controls RNA of select Transposable Elements (*TE*s). Here we show that in Zfrp8/PDCD2 knock down (KD) ovaries, specific mRNAs and *TE* transcripts show increased nuclear accumulation. We also show that Zfrp8/PDCD2 interacts with the (40S) small ribosomal subunit through direct interaction with RpS2 (uS5). By studying the distribution of endogenous and transgenic fluorescently tagged ribosomal proteins we demonstrate that Zfrp8/PDCD2 regulates the cytoplasmic levels of components of the small (40S) ribosomal subunit, but does not control nuclear/nucleolar localization of ribosomal proteins. Our results suggest that Zfrp8/PDCD2 functions at late stages of ribosome assembly and may regulate the binding of specific mRNA-RNPs to the small ribosomal subunit ultimately controlling their cytoplasmic localization and translation.

## Introduction

PDCD2 is an evolutionarily conserved eukaryotic protein, that is required in stem- and embryonic cells of many different organisms and also facilitates cancer cell growth [1–5]. It is essential in mouse embryonic stem cells and *PDCD2* knock out embryos die prior to implantation [2, 4]. In acute leukemia patients PDCD2 expression correlates with disease status and is a significant predictor of clinical relapse [3].

In *Drosophila*, loss of function of the fly PDCD2 homolog Zfrp8 causes gradual lethality between 1st instar larval and pharate adult stages, accompanied by strong abnormalities in the major larval hematopoietic organ, the lymph gland [6]. Z*frp8/PDCD2* is essential for the maintenance of hematopoietic stem cells in *Drosophila* [7] and also in follicle and germline stem cells (GSCs) in the ovary [8]. Lack of Zfrp8 causes an arrest of stem cell division and ultimate loss of the follicle and germline lineages. All phenotypes can be rescued by the expression of human PDCD2, demonstrating that the molecular function of Zfrp8/PDCD2 is conserved [8].

In addition to strong GSC phenotypes, *Zfrp8* KD ovaries show abnormal distribution of the Bic-D and Orb oocyte specification factors in the germarium, nurse cells, and within the oocyte, phenotypes that are also observed in piRNA pathway mutants [8–13]. Like piRNA pathway proteins, Zfrp8 has a repressive effect on Transposable elements (*TEs*), however it only controls the expression of select *TE*s [8]. *Zfrp8* has weaker effects on *TE* levels than any piRNA pathway mutant, but shows stronger phenotypes, especially in stem cells. Zfrp8 may therefore also regulate genic transcripts that are particularly important for stem cell maintenance or cell proliferation.

The mislocalization of BicD and Orb as well as the severe abnormalities of the *Zfrp8* germ line may result from abnormal localization of their mRNA and defects in spatial translation, suggesting that Zfrp8/PDCD2 may be immediately involved in mRNA transport. Our recent studies show that PDCD2/Zfrp8 interacts with a number of proteins responsible for mRNA localization and translational repression [14] such as FMRP/Fmr1 (Fragile-X Mental Retardation Protein [15], and NUFIP1/Nufip (Nuclear FMRP-interacting Protein, [14, 16]). FMRP has been shown to associate with NUFIP and with mRNAs in the nucleus and this RNP complex is exported into the cytoplasm, where FMRP regulates mRNA translation and degradation [17–19]. *Zfrp8* directly binds Nufip, is required for the proper localization of FMRP to cytoplasmic puncta, and likely affects the same or overlapping RNA/mRNA targets as FMRP [14]. The genetic interaction between *Fmr1* and *Zfrp8* shows that they have antagonistic functions [14].

Recently, Burroughs and Aravind [20] proposed that PDCD2/Zfrp8 belongs to a large family of TYPP domain (TSR4, *Y*wqG, PDCD2L, and PDCD2) proteins that contains both eukaryotic and prokaryotic members [20]. TSR4, the yeast member of this family, albeit biochemically uncharacterized, has been reported to function in rRNA and ribosome maturation [21]. *Drosophila* and vertebrates encode two members of the family, Trus/PDCD2L containing an intact TYPP domain and Zfrp8/PDCD2 in which the TYPP domain is interrupted by MYND (Myeloid, Nervy, and DEAF-1), another protein-protein interacting domain [20, 22]. Therefore, while it is possible that both Trus/PDCD2L and Zfrp8/PDCD2 play a role in eukaryotic ribosome maturation, Zfrp8/PDCD2 may have lost some TYPP domain-specific activities and at the same time acquired additional functions.

The assembly and maturation of eukaryotic ribosomes involves different compartments of the cell, and is regulated by multiple non-ribosomal factors (for review see [23–25]). Most ribosomal proteins are synthesized in the cytoplasm and imported into the nucleus. The assembly of both ribosomal subunits starts in the nucleolus on polycistronic pre-rRNA transcripts (in *Drosophila*, 18S, 5.8S, 2S and 28S). The maturation of the (small) 40S subunit requires cleavage of pre-rRNA between 18S and 5.8 rRNA [23, 25–27]. There, the mature 40S subunit binds mRNA that in turn facilitates association of the 40S and 60S ribosomal subunits and initiation of translation. The stability of individual ribosomal proteins dramatically increases upon each step of ribosome maturation including assembly into subunits, export from the nucleus, and binding to mRNA [28–30].

The 40S ribosomal subunit plays an important role in the initiation and fidelity of translation. It consists of more than 30 proteins forming a conserved core and a eukaryotic-specific “shell" that ensures binding to multiple non-ribosomal proteins, which regulate subunit assembly, nuclear-cytoplasmic transport, and specificity of translation initiation [31–33]. One of the 40S components, RpS2 (uS5 in the new nomenclature of ribosomal proteins [34]) is related to the Escherichia coli S5 ribosomal protein, but has N- and C- terminal eukaryotic-specific extensions [31, 35]. The N terminus of RpS2 directly interacts with and is methylated by protein arginine methyltransferase 3 (PRMT3/Rmt3 [36–39]). This interaction affects general stability of RpS2 and may also regulate the binding fidelity between 40S and mRNA. Other regulatory, eukaryotic functions of RpS2 include pre-40S export competence and transport of 40S precursors from the nucleus to the cytoplasm [40].

In our study we show that in *Zfrp8* KD cells, the cytoplasmic levels of RpS2 and at least two other 40S components, RpS11 (uS17), and RpS13 (uS15), are reduced, suggesting that Zfrp8 may regulate their nuclear export, export competency, or even the final cytoplasmic maturation steps that include mRNA binding and 60S-40S assembly. Because of the reduction of RpS2 levels in *Zfrp8* KD cells we anticipated that Zfrp8 may have a strong effect on general translation. However, the expression of many proteins was maintained at relatively normal levels, suggesting that lack of Zfrp8 affects translation in a transcript specific manner.

We tested the possibility that Zfrp8/PDCD2 regulates processing of pre-rRNA, mRNA or piRNA and did not find any evidence supporting a direct role of the protein in RNA cleavage or splicing. Instead we found that Zfpr8/PDCD2 is required for efficient nuclear export of select transcripts, including some *TE-RNAs* and endogenous mRNAs. Based on the predicted chaperone activity of Zfrp8/PDCD2, its interaction with RNA binding proteins [8, 14], we propose that Zfrp8 assists the assembly of transcript-specific RNPs and facilitates their nuclear export. We further propose that Zfrp8/PDCD2 regulates the binding of these RNPs to the small ribosomal subunit, thereby providing selective translational control.

## Results

### Zfrp8/PDCD2 interacts with ribosomal protein RpS2

**Fig 1:**
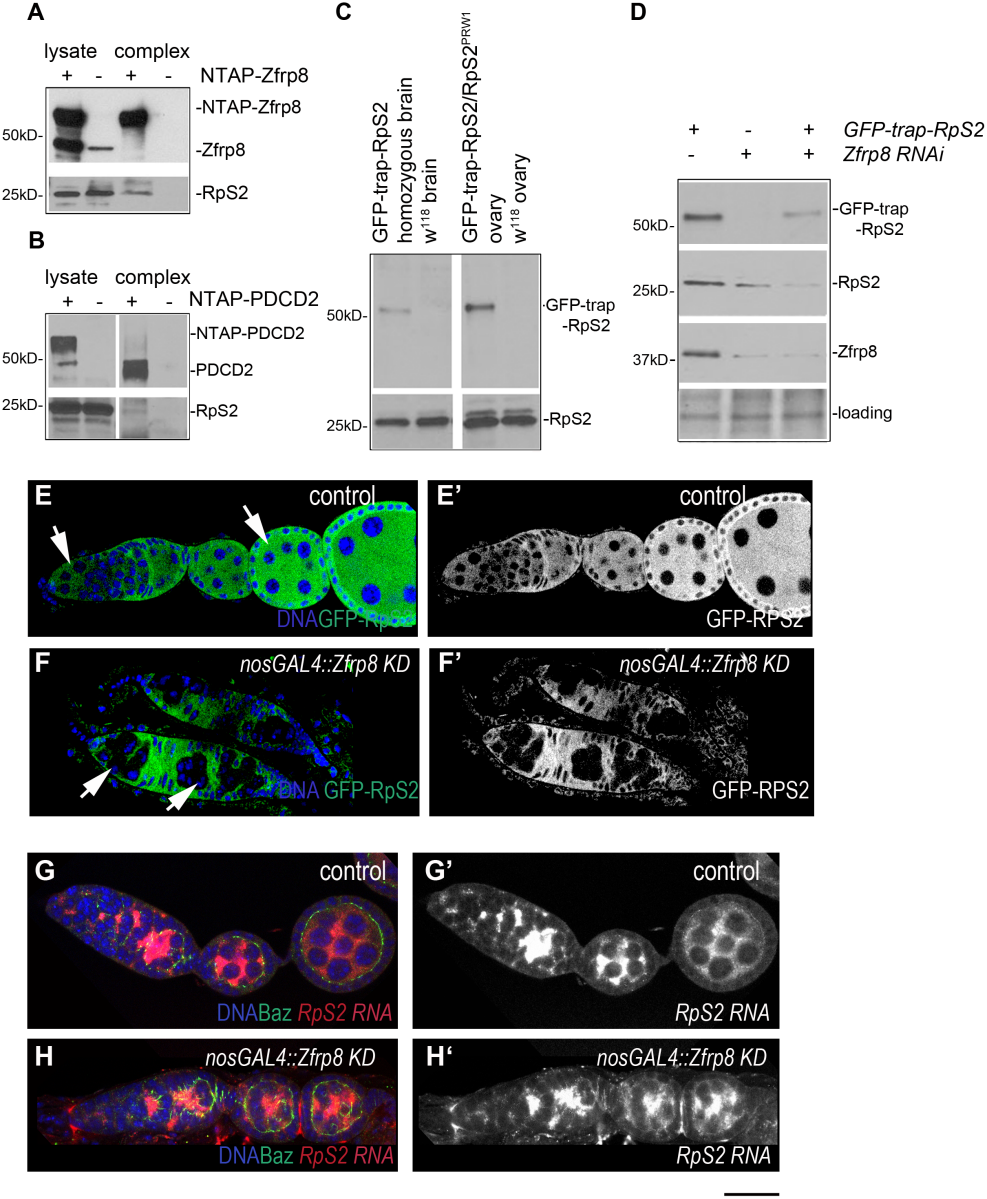
Zfrp8/PDCD2 complexes with RpS2 and controls its levels or stability. (A) Western blot of the TAP-Zfrp8 complex affinity purified from Drosophila ovaries (*Zfrp8*^*null*^, *da-GAL4/UAS-TAP-Zfrp8*) was probed with anti-Zfrp8 (top) and anti-RpS2 (bottom) antibodies. Extracts from *w*^*118*^ flies was used as negative control. Only small portion of RpS2 was co- purified with Zfrp8, and therefore longer exposure was needed to detect RpS2 in Zfrp8 complex. (B) Western blot of the TAP-PDCD2 complex affinity purified from HEK293 cells was probed with anti-PDCD2 (top) and anti-RpS2 (bottom) antibodies. Cells transfected with vector alone (NTAP) were used as negative control. (C) GFP-trap-RpS2 homozygous flies expressed both, tagged protein (anti-GFP, top), and more abundant untagged RpS2 protein (anti-RpS2, bottom). (D) Western blot of *Zfrp8* KD larval brains; both GFP-trap-RpS2 and endogenous RpS2 were reduced (lane 2 and 3) compared to that in control (lane1). The blot was probed with anti-GFP, anti-RpS2, and anti-Zfrp8 antibodies. Ponceau S was used for loading control. (E-E') GFP-trap-RpS2 protein (green) was present in germ line (arrows) and somatic ovarian cells and was mainly localized to the cytoplasm. In *Zfrp8* KD germ line (F, F', arrows) GFP-trap-RPS2 was significantly reduced. (G-H') FISH with RpS2 probe (red) showed no difference in *RpS2* transcript level and distribution between *Zfrp8* KD (H-H') and control ovaries (G-G'). Ovaries were co-stained with DAPI (DNA, blue) and anti-Bazuka antibody (green). Size bar is 20μm.

In order to gain insight into the molecular function of Zfrp8 and identify proteins that directly associate with Zfrp8 we performed a small scale two hybrid screen using normalized *Drosophila* universal library (S1 Table) and identified 5 independent clones encoding S2 component of the 40S ribosomal subunit (RpS2, S1 Table). To confirm this interaction *in vivo*, we performed TAP purification of NTAP-Zfrp8 from *Drosophila* ovaries and NTAP-PDCD2 from HEK293 human cells, and showed that Zfrp8/PDCD2 interacts with RpS2 in flies and in human cells (Fig 1A and 1B). The interaction between RpS2 and Zfrp8/PDCD2 is also reported for several protein interaction networks and two-hybrid screens from different organisms [41–43]. RpS2 was also the most abundant Zfrp8 partner that we recently identified by tandem affinity purification (TAP) followed by mass spectrometry (224 peptides, [14]). Importantly, the TAP purification also identified additional components of the 40S subunit (RpS3, RpS4, RpS5a and RpS7, 11–38 peptides, [14]) indicating that Zfrp8/PDCD2 may interact with RpS2 as part of the subunit.

In *Drosophila*, *RpS2*^*PRW1*^, the strongest loss of function allele, causes lethality at first instar larval stage, while a P-element insertion 28nt upstream from the transcription start of *RpS2* (*RpS2*^*P*^ also called *sop*^*P*^) is homozygous viable and causes female sterility. *RpS2*^*P*^ homozygous and hemizigous (*RpS2*^*P*^*/RpS*^*PRW1*^) ovaries have reduced levels of RpS2 protein and show the "string of pearls" (*sop*) phenotype; all egg chambers arrest development at stages two or three ([44] and S1A–S1D and S1H Fig). Upon close inspection of these ovaries we detected abnormalities in GSC divisions, abnormal spectrosomes, and a delay in egg chamber growth (S1C–S1H Fig). This phenotype is similar to the phenotype observed when Zfrp8 is depleted in the germline either by induction of *Zfrp8* clones or germline specific KD [8].

In addition to these similarities in phenotype and the physical interaction between the two proteins, *Zfrp8* and *RpS2* showed strong genetic interaction. The Zfrp8 KD ovary phenotype was dominantly enhanced by *RpS2*^*P*^ and *RpS2*^*PRW1*^ (S1I, S1K and S1L Fig). Also the *RpS2*^*P*^ phenotype was enhanced in a *Zfrp8* heterozygous background *(Zfrp8*^*null*^*/+*, S1H and S1J Fig), suggesting that the two proteins function synergistically, at least during early oogenesis.

### RpS2 cellular levels depend on Zfrp8

We further investigated if *Zfrp8* and *RpS2* controlled each other, and found that Zfrp8 showed normal levels and distribution in *RpS2*^*P*^ ovaries (S1A–S1D Fig). To assess if *Zfrp8* controls RpS2 we took advantage of the GFP-trap-RpS2 line that contains the GFP-coding sequence flanked by splice acceptor and donor between first and second exons of *RpS2* [45, 46]. The GFP distribution of trap-lines reflect endogenous patterns of protein expression and can be visualized with minimum tissue processing. GFP-trap-RpS2 line expresses low but visible amounts of N terminally GFP tagged RpS2 and also untagged wild type RpS2, likely resulting from alternative splicing (Fig 1C, [46, 47]), and is homozygous viable and fertile.

In control ovaries, GFP-trap-RpS2 was present in all cells (Fig 1E and 1E'), and showed mostly cytoplasmic distribution. When GFP-trap-RpS2 was combined with *nos-GAL4*, *Zfrp8 RNAi* (*Zfrp8 KD*), GFP-trap-RpS2 remained at normal levels in somatic cells, but was strikingly decreased in the *Zfrp8* germ line cells (Fig 1F and 1F', arrows).

We confirmed that Zfrp8 affected both the tagged and the untagged RpS2 by western blotting in brain extracts from Zfrp8 KD larvae (see Materials and Methods, Fig 1D). We further tested *RpS2* mRNA levels in control and *Zfrp8* KD ovaries by qRT-PCR and by *in situ* hybridization (Figs 1G–1H' and 4A) and found that Zfrp8 does not affect *RpS2* transcript levels. These results indicate that Zfrp8 regulates RpS2 posttranscriptionally.

### Zfrp8 controls cytoplasmic levels of several components of the small ribosomal subunit

The GFP-trap line produced different forms of RpS2 (Fig 1C), and it also enhanced *Zfrp8 KD* phenotype (Fig 1B). We, therefore constructed a transgenic line expressing mCherry-RpS2 (UAS(P)-mCherry-BD-RpS2, Materials and Methods) and also used a fly line expressing a GFP-tagged RpS2 (UAS(T)-GFP-RpS2 [48, 49]) for our experiments. When expressed in the germ line (*nos-GAL4* driver), mCherry-RpS2 mirrored the expression pattern of the driver, showing highest levels in the cytoplasm of GSCs and egg chambers after stage 7 (S2A, S2B and S2I, S2J Fig). In *Zfrp8 KD* germ line (*nos-GAL4*, *UAS- mCherry-BD-RpS2/UAS-Zfrp8 RNAi)* the levels of mCherry-RpS2 were dramatically reduced (S2I–S2L Fig). Because the transcription and translation of both *GFP-RpS2* and *mCherry-RpS2* are controlled by GAL4/UAS and SV40 3'UTR our results confirm that Zfrp8 regulates RpS2 at the post-transcriptional and likely post-translational levels. Since the proteins interact directly we propose that Zfrp8 may stabilize RpS2.

To ensure that *Zfrp8* KD does not block *nos-GAL4*-driven expression in general, we tested the expression of another N-terminally tagged ribosomal protein, GFP-RpS18 [48, 49], and the expression of Venus (a fast maturing fluorescent protein [50, 51]), driven by *nos-GAL4* in wild type and *Zfrp8* KD backgrounds. No reduction in the levels of Venus or RpS18 were detected, however, both proteins showed atypical distribution patterns in *Zfrp8* ovaries, consistent with the developmental abnormalities observed (compare A-B and C-D, E-F and G-H in S2 Fig).

**Fig 2:**
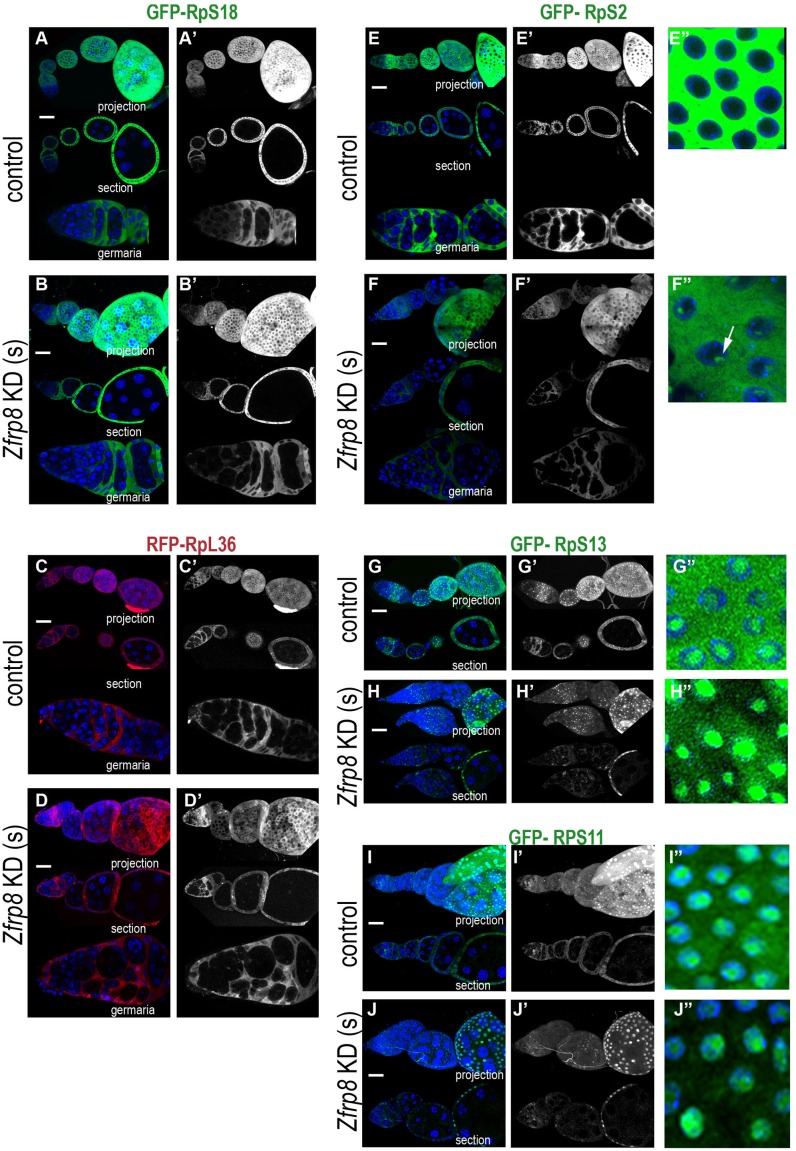
Zfrp8 regulates cytoplasmic levels of RpS2, RpS11 and RpS13 in somatic cells. (A-B') *tj-GAL4* driven GFP-RpS18 expression shows similar levels and distribution in control (A-A') and *Zfrp8* KD(s) follicle cells. (C-D’) Similarly, RFP-RpL36 (red) levels are not changed in *Zfrp8* KD(s) follicle cells (D-D’) compared to controls (C-C’). (E-F") *Zfrp8* KD causes the reduction in cytoplasmic levels of GFP-RpS2 (green) in the follicle cells(F-F‴). Compare the protein levels in germaria and consecutive egg chambers to similar stages in control (E-E"). Lack of Zfrp8 also cause accumulation of GFP-RpS2 in nuclear clusters (arrow, compare E" and F"). (G-J") GFP-RpS13 (green, G-H") and GFP-RpS11 (green, I-J") were localized in the cytoplasm and nucleoli in follicle cells of control ovaries (G-G", I-I"). Cytoplasmic levels of both proteins were reduced in *Zfrp8* KD follicle cells, however the protein accumulation in nuclei and nucleoli was not affected (compare H" and G", J" and I"). DAPI (DNA, blue), size bar is 20μm.

Null mutation in *Zfrp8* causes loss of follicle stem cells (FSCs), while knock down of *Zfrp8* in somatic cells (*Zfrp8* KD(s) of the ovary causes an arrest in egg chamber development at stages 7–9 [8]. Therefore we investigated if *Zfrp8* also affected RpS2 and RpS18 levels in the soma and expressed the *UAS-GFP-RpS2* and *UAS-GFP-RpS18* transgenes in the somatic cells of the ovary under the control of the *traffic jam (tj)-GAL4* driver. We compared the levels and distribution of GFP-RpS2 and GFP-RpS18 in somatic cells in germaria and egg chambers of *Zfrp8* KD(s) and control ovaries. Similar to what was observed in the germ line, in *Zfrp8* KD(s) cells, RpS18 levels were unchanged while RpS2 was significantly reduced in somatic cells both in germaria and egg chambers (compare similar stages in Fig 2A–2B' and 2E–2F"). Interestingly, in later stage egg chambers (7–8), loss of Zfrp8 caused accumulation of RpS2 in nuclei, where it formed perinucleolar clusters (see arrow in Fig 2F").

To check if Zfrp8 affects more than one ribosomal protein we tested 9 other transgenic lines that express GFP or RFP tagged ribosomal protein genes (see Table 1, [48]). We identified two additional components of the small (40S) ribosomal subunit, RpS13 and RpS11 that were significantly changed in the *Zfrp8* KD(s) background. Both proteins when expressed under *tj-GAL4* were seen in the cytoplasm and in nucleoli of wild type follicle cells (Fig 2G–2G" and 2I–2I"). In *Zfrp8* KD(s) cells, the cytoplasmic levels of RpS13 and RpS11 were dramatically reduced at all developmental stages, from germarium to egg chambers stages 7–9, but the levels of both proteins in nuclei and nucleoli of follicle cells were similar to that in the control (Fig 2G–2J"). In contrast, the distribution of two components of the large ribosomal subunit (60S), RpL8 (uL2) and RpL36 (eL36), that also show cytoplasmic and nucleolar localization, were not affected in *Zfrp8* KD(s) (Fig 2C–2D', and not shown). Furthermore, the amounts of two additional ribosomal proteins, RpL23 (uL14) and RpS15 (uS19), that are mainly localized to the nucleus [48, 52] were not reduced in *Zfrp8* KD(s) cells. Thus, lack of Zfrp8 specifically affected the cytoplasmic stability of at least three components of the 40S ribosomal subunit (Table 1), but did not disrupt their nuclear/nucleolar localization, suggesting that Zfrp8 regulates the maturation of the 40S subunits at pre-40S assembly in nucleoli, its nuclear export, or its final assembly into ribosomes, rather than the expression, stability and nuclear import of individual ribosomal proteins.

**Table 1:** Effect of Zfrp8 on Ribosomal proteins.

not changed in *Zfrp8* KD	insignificantly changed in *Zfrp8* KD	strongly reduced in the cytoplasm of *Zfrp8* KD cells
RpS18(uS13), RpL8(uL2), RpL36(eL36), RpL23(uL14, nuclear), RpS15(uS19, nuclear)	RpL32(eL32), RpS5a, RpS9(uS4)	RpS2(uS5), RpS11(uS17), RpS13(uS15)

UAS constructs of GFP tagged RpS2, RpS11, RpS13, RpS5a, RpS15, RpS18, RpS9 and RFP- tagged RpL23, RpL32, RpL36, RpL8 [48, 49] were expressed in follicle cells (*tj-GAL4*) in control and Zfrp8 KD(s) ovaries. UAS(P)-mCherry-BD-RpS2, UAS(P)-GFP-RpS18 express in both somatic and germ line cells and were tested with *nos*-GAL4 driver in control and *Zfrp8* KD ovaries.

### Zfrp8 and general translation

A possible consequence of the reduced cytoplasmic levels of RpS2 and other components of the 40S subunit observed in *Zfrp8 KD* tissues, could be a decrease of general protein synthesis. However, a number of transgenic proteins, including GFP-RpS18, RFP-RpL36, Venus, or GAL4, showed overall normal protein expression levels in *Zfrp8* KD tissues (Fig 2 and S2 Fig). We tested levels and distribution of 17 additional GFP tagged proteins with different turnover rates that are involved in various molecular processes. GFP protein trap lines allowed us to monitor the pattern of protein expression in germ line and somatic cells at different stages of oogenesis and to compare the protein levels and distribution in germaria and early egg chambers of *Zfrp8* KD and control ovaries (S2 Table, S3 Fig, [45, 46]). 15 out of the 17 proteins showed the same levels of expression and similar distribution in germaria and early egg chambers of *Zfrp8* KD and of controls. S3 Fig shows the expression patterns of Zn72D (nuclear, S3A–S3D Fig), Me31B (cytoplasmic, S3E–S3H Fig) and Cyclin B (Cyc B, S3I–S3L Fig) in control and *Zfrp8* KD backgrounds. CycB represents an important example of a protein with high turnover rate. It is dynamically expressed in dividing follicle cells (S3I–S3L Fig), germ line stem cells, and cystoblasts (arrows). In *Zfrp8 KD* ovaries, the numbers of germ line cells expressing CycB and the levels of expression were not significantly different from those in controls (S3I–S3L Fig, arrows).

Interestingly, two translation factors, Eukaryotic initiation Factor 4E (EiF4E, S3M–S3P Fig), and Elongation Factor 2 (EF2, S3Q–S3T Fig), were also present at normal levels in *Zfrp8 KD* germaria (arrows), but showed visible reduction in egg chambers (arrowheads), correlating with *Zfrp8* KD developmental defects. Overall, our results argue against a general requirement of Zfrp8/PDCD2 in translation, but they point to a connection of Zfrp8 with the translational machinery and a role of Zfrp8 in regulating gene-specific protein production.

### Transcript specific functions of Zfrp8/PDCD2

The gene specific functions of PDCD2/Zfrp8 are also supported by prior observations. Zfrp8 associates with Mael and genetically interacts with multiple components of piRNA pathway, but only controls the expression of select *TE*s ([8] and paper in preparation). Further, Zfrp8/PDCD2 functions with Nufip and FMRP and therefore may regulate the nuclear export or localization of FMRP-dependent mRNAs [14].

To test if Zfrp8 controls subcellular localization of specific transcripts, we performed fluorescent *in situ* hybridization (FISH) using probes specific for the *Pino*, *sta*, *RpS2* and *RpL36* genes, and the *TAHRE* tansposon (see Materials and Methods). *TAHRE* was chosen because of its low copy number in the *Drosophila* genome and because it was strongly de-repressed in *Zfrp8 KD* ovaries (*log2(fold)>7 p*,<*10*^−*2*^, manuscript in preparation). The two genes, *Pino* and *sta* were also identified as potential targets of *Zfrp8* regulation (manuscript in preparation).

**Fig 3:**
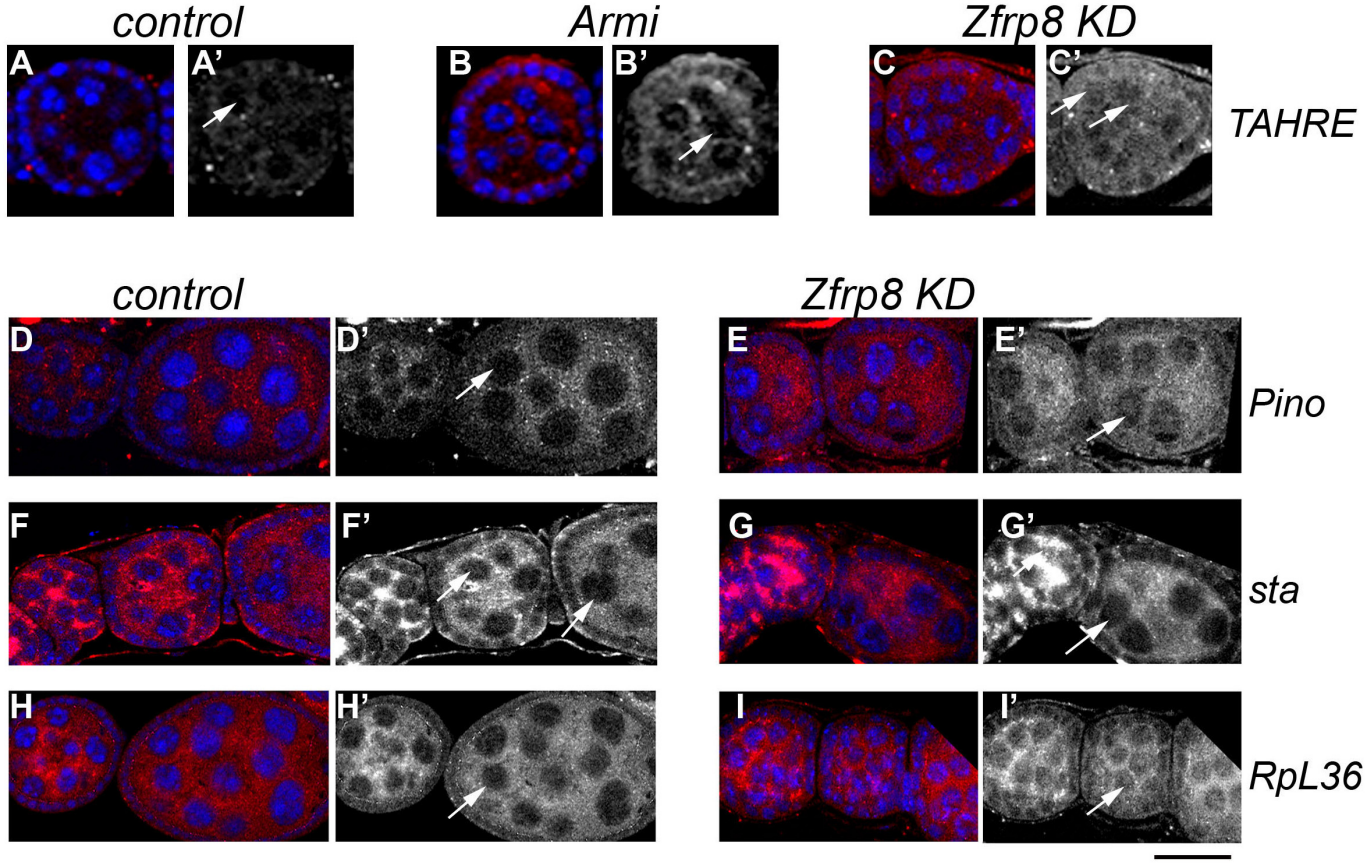
Zfrp8 affects cytoplasmic localization of select transcripts. (A-C') Levels and localization of *TAHRE* transcripts (FISH, red) were low in control ovaries (A-A"), and strongly increased in the cytoplasm of *Armi*^*1*^*/Armi*^*72*.*1*^ ovaries (B-B', arrow). In *Zfrp8 KD* ovaries *TAHRE* RNA was present at similar levels in both nuclei and cytoplasm (D-D', arrows). (D-I') FISH with *Pino* (D-E), *sta* (F-G') and *RpL36* (H-I") probes. Levels of *Pino* RNA is somewhat increased in *Zfrp8 KD* ovaries (E-E'), and significantly elevated in nurse cell nuclei (arrows in D' and E'). (F-G') *sta* transcripts did not show changes in levels or localization in *Zfrp8* KD. (H-I') *RpL36* transcript showed increased nuclear accumulation in *Zfrp8* KD ovaries (arrows, in H' and I'). Additional images of ovarioles including germaria are shown in S4 Fig. DNA blue (DAPI), size bar is 20μm.

In driver only control ovaries, *TAHRE-RNA* was present at very low levels (Fig 3A and S4A and S4B Fig). We also used ovaries derived from the piRNA pathway mutant *Armi*, because TE transcripts including *TAHRE*, are increased in this mutant (Fig 3B and 3B' and S4C and S4D Fig, arrows). While in Armi ovaries *TAHRE* RNA was mostly cytoplasmic, in *Zfrp8* KD ovaries it was more uniformly distributed in both nuclei and cytoplasm (Fig 3C and 3C', S4E and S4F and S5A Figs), suggesting that the nuclear export of *TAHRE* RNA may be impaired. Similar changes were observed for *Pino* transcripts, which showed increased accumulation in *Zfrp8* KD nuclei compared to that in control (Fig 3D–3E', S4G–S4J Fig, arrows and S5B Fig). Therefore, in the absence of Zfrp8, at least some of the *TAHRE* and *Pino* transcripts accumulated in the nucleus.

**Fig 4:**
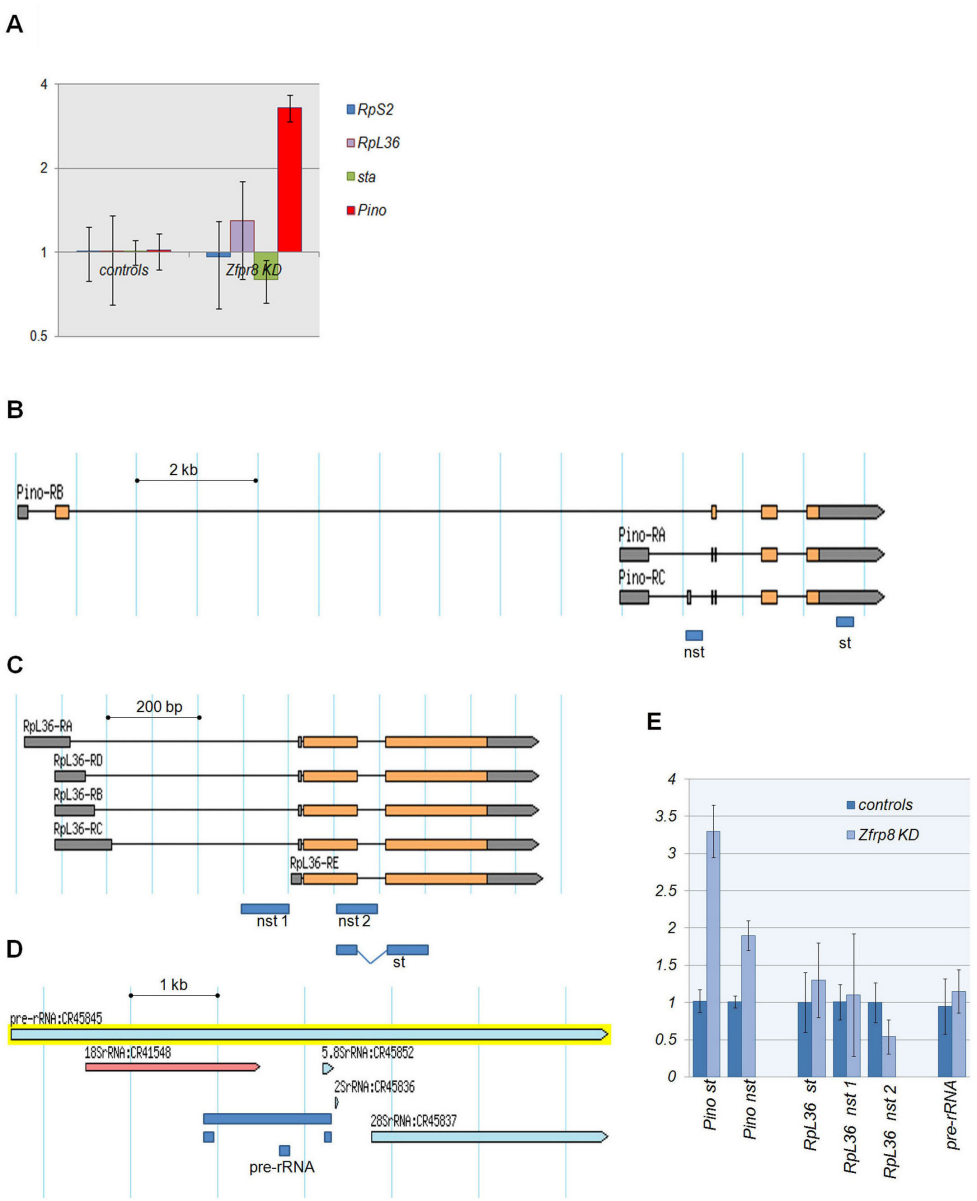
Gene expression and RNA processing in *Zfrp8* KD ovaries. (A) Quantitative RT-PCR of fold accumulation of select transcripts in young ovaries. (B-D) Exon-intron structures of *Pino* and *RpL36* loci and typical structure of pre-rRNA (according to FlyBase). Fragments/primer pairs used for qRT-PCR are show as navy blue rectangles. Primer pares are listed in S3 Table. (E) qRT-PCR of total RNA from control and *Zfrp8* KD ovaries showed no accumulation of non-spliced transcripts (nst) and un-processed pre-rRNA. Fragments for spliced transcripts are labeled "st". (A and E) Fold accumulation of the transcripts in controls (*nos-GAL4*/+ and *UAS-Zfrp8 RNAi*/+) and in *Zfrp8* KD (nos-*GAL4/UAS-Zfrp8 RNAi*) are shown relative to *w118* controls (mean ± SD; n≥ 3, normalized to *Rp49/RPL32*,*GAPDH2*,*and RpS2*).

No changes in *sta* RNA distribution were observed in *Zfrp8 KD* ovaries (Fig 3F–3G', S4K–S4N and S5C Figs). We also compared the levels and distribution of RNAs of the two ribosomal proteins, RpS2 and RpL36, in control and *Zfrp8* KD ovaries. While *RpS2* transcripts showed no visible changes (Figs 1E–1F', 4A and S5E Fig), *RpL36* transcripts were enriched in *Zfrp8* KD nuclei (Fig 4H–4I', S4O–S4R and S5D Figs), but the overall levels were not increased (Fig 4A and S5D Fig). Together these results confirm that Zfrp8/PDCD2 regulates a subset of transcripts, from both *TE*s and protein coding genes, likely by facilitating their nuclear export.

Because Zfrp8/PDCD2 is structurally related to TSR4, a protein involved in pre-rRNA cleavage and 18S r-RNA maturation in yeast [20, 21], it has been suggested that Zfrp8/PDCD2 may have similar activity in higher eukaryotes, facilitating the processing of rRNAs or splicing of mRNAs. We tested if *Zfrp8 KD* caused accumulation of non-spliced *RpL36* and *Pino* transcripts, or of pre-rRNA (Fig 4B–4E). We did not detect any effect of *Zfrp8* on the relative levels of precursor RNAs (Fig 4E), neither could we demonstrate any complexing of Zfrp8/PDCD2 with RNAs (ribonucleoprotein-IP (RIP, [53]), data not shown). The change of activity may be explained by the structure of the Zfrp8/PDCD2. In contrast to the TSR4 and PDCD2L, the TYPP domain in Zfrp8/PDCD2 is interrupted by MYND another protein-protein interaction domain [20, 22]. Thus our results are consistent with the idea that Zfrp8/PDCD2 instead of RNA processing has acquired other activities and functions as a chaperone in ribosome assembly or may also regulate mRNA localization and translation.

## Discussion

We have found that Zfrp8/PDCD2 interacts with RpS2 in a number of assays, including the yeast two-hybrid, consistent with direct interaction between the two proteins. While non-specific binding of bait proteins to ribosomal proteins are always a concern, the genetic interaction between *Zfrp8/PDCD2* and *RpS2* and strong effect of *Zfrp8* on cytoplasmic levels of RpS2 confirms the validity of the interaction between the two proteins.

RpS2 is a component of the small ribosomal subunit and may bind Zfrp8/PDCD2 as an individual protein or as part of the subunit. We previously identified several 40S ribosomal proteins (RpS2 (uS5), RpS3 (uS3), RpS4 (eS4), RpS5a and RpS7 (eS7) as part of the Zfrp8 complex [14]. Knock down of Zfrp8 also affects the cytoplasmic levels of several 40S components (RpS2 (uS5), RpS11 (uS17), and RpS13 (uS15)). Based on these data, we propose that Zfrp8/PDCD2 interacts with the small ribosomal subunit rather than with free RpS2. However, it is not clear if Zfrp8 interacts with the partially assembled or the mature 40S subunit. Interestingly, *Zfrp8* KD does not affect the stability of all RpS proteins, for instance RpS18 (uS13) and RpS15 (uS19) remain unaffected by loss of Zfrp8 (Fig 2 and S2 Fig, Table 1), but then these ribosomal proteins may be more stable than others when not assembled into the subunit.

Like most ribosomal proteins, RpS2 is synthesized in the cytoplasm, transported into the nucleolus, where it is required for several steps of ribosome maturation: assembly of ribosomal proteins on pre-rRNA, pre-rRNA cleavage, nuclear export of the competent pre-40S subunit, its cytoplasmic maturation, interaction of the small subunit with mRNA and assembly into the ribosome [38, 40]. At each of these steps the stability of individual ribosomal proteins greatly increases, and Zfrp8/PDCD2 may control one or more of these steps. In normal ovaries, tagged RpS2 was undetectable in nuclei and nucleoli (Figs 1E and 2E–2E"), but it showed visible nuclear accumulation in *Zfrp8* KD(s) follicle cells (Fig 2F–2F"). Furthermore, the relatively high nuclear and nucleolar levels of two other ribosomal proteins, RpS11 and RpS13, were not changed in *Zfrp8* KD(s) cells (Fig 2G–2J'), but the cytoplasmic levels of all three proteins were dramatically reduced. This suggests that Zfrp8/PDCD2 does not affect the synthesis of ribosomal proteins nor their nuclear import, but that it functions later, in ribosome assembly in the nucleolus, or nuclear export of the 40S subunit, or it may stabilize the small ribosomal subunit in the cytoplasm.

Because pre-rRNA cleavage and 18S rRNA trimming are essential steps in 40S maturation [36, 40, 54] we tested if *Zfrp8* has an effect on rRNA processing. We did not observe any significant accumulation of -rRNA precursors in *Zfrp8* KD ovaries. This result is similar to what was observed in *PDCD2* KO mouse embryonic fibroblasts, where pre-rRNAs were not increased [4] and (Rabson A. personal communication). These results indicate that Zfrp8/PDCD2 functions after pre-40S assembly and pre-rRNA cleavage, possibly at the level of 40S subunit nuclear export or its final assembly into the mature ribosome in the cytoplasm.

The apparent reduction of 40S subunit components in the cytoplasm of *Zfrp8* KD cells does not lead to a universal block of translation as many proteins are being produced at relatively normal levels. Further, *Zfrp8* mutant and KD phenotypes as well as PDCD2 KO and KD in mouse and human cells [2–4] show that Zfrp8/PDCD2 is essential in stem cells and highly proliferative cells, but has little or no function in differentiated cells and cells with low proliferative activity. Several explanations for these cell- or tissue-specific phenotypes are possible. One, highly proliferative and stem cells, requiring high levels of protein synthesis, are especially sensitive to levels of ribosomes. For instance, the decrease in ribosomal biogenesis may trigger premature stem cell differentiation. This phenotype, was observed in *wcd* mutants (*wicked* encodes U3 snoRNP associated protein) and may result not only from general abnormalities in rRNA maturation, but also from defects in asymmetric segregation of ribosomal biogenesis factors [55]. Two, the lack of Zfrp8/PDCD2 may cause an imbalance of ribosomal proteins that are not assembled into subunits and this is detrimental to stem- and highly proliferative cells. In this context it is interesting to note that select unbound ribosomal proteins inhibit MDM2 E3 ligase activity, cause p53 stabilization, and cell cycle arrest [56–58]. Therefore, imbalance of ribosomal proteins may explain the cell cycle arrest and marked increase of nuclear p53 observed in *PDCD2* KO MEFs, ESCs, and embryonic blastocysts [4].

A third explanation could be that, Zfrp8/PDCD2 functions in regulating select transcripts essential in stem- and proliferating cells. We identified several *TE* transcripts and protein coding mRNAs that are selectively regulated by Zfrp8 in the germ line [8] and paper in preparation). We tested the subcellular localization of one of the *TE*s (*TAHRE)* that is de-repressed in *Zfrp8* KD ovaries and found that *TAHRE* transcript levels were not only increased, but also showed uniform nuclear-cytoplasmic distribution in *Zfrp8* KD ovaries. This effect is different from what is observed in the majority of piRNA pathway mutants, where *TE* (e.g. *TAHRE)* transcripts are predominantly seen in the cytoplasm (Fig 3C and 3C', [59]). Therefore, we propose that Zfrp8 may facilitate export of *TAHRE* RNA from the nucleus as well as its targeting to the cytoplasmic sites of RNA processing and degradation. Similarly, *Zfrp8* affects the transport of select protein coding mRNAs from the nuclei to proper cytoplasmic locations. The mRNA of two genes *Pino* and *RpL36*, showed visible nuclear accumulation in *Zfrp8* KD ovaries. *Pino* also showed some increase in transcript level while *RpL36* mRNA levels were unchanged (S4A Fig). We tested if nuclear accumulation of *Pino* and *RpL36* transcripts was associated with inefficient splicing. We did not detect any accumulation of non-spliced RNAs and therefore propose that Zfrp8/PDCD2 regulates nuclear export and post-export localization of mature transcripts, steps occurring post splicing. Therefore, Zfrp8 may influence not only degradation but also the efficiency and the spatial control of their translation.

Zfrp8/PDCD2 is distributed in the cytoplasm and nuclei of most cells and while required in nuclei, may have an important function in both cellular compartments [2, 8, 14]. Zfrp8/PDCD2 could control the formation of specialized RNA binding complexes or RNPs co-transcriptionally by recruiting different RNA binding proteins (NUFIP/FMRP, MAEL/PIWI, Hrb27C) and thereby regulate the fate of various transcripts [8, 14].

**Fig 5:**
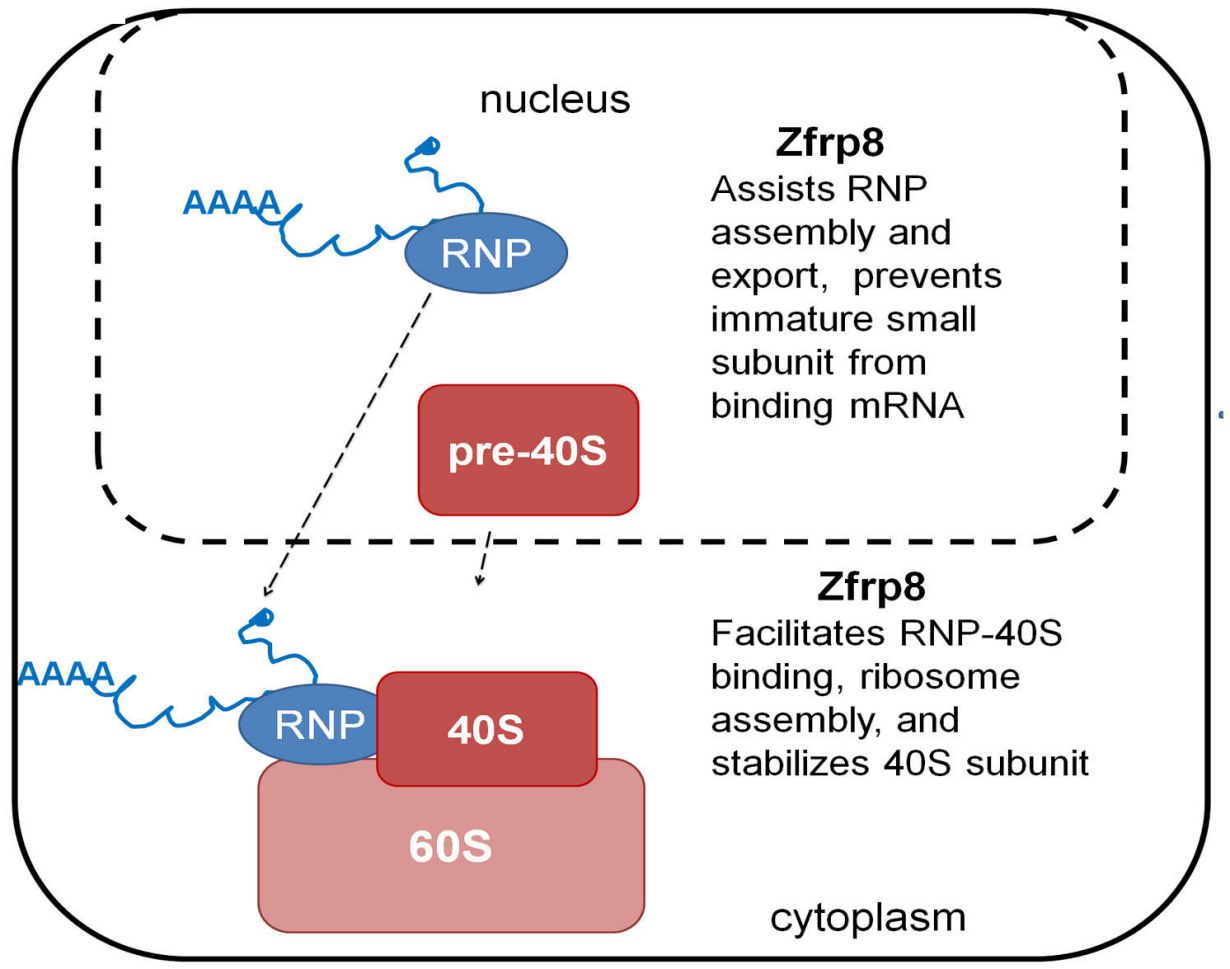
Graphic model of Zfrp8/PDCD2 function.

In *Zfrp8* KD cells, we observed both mis-regulation of select transcripts and ribosomal abnormalities. While it is possible that Zfrp8/PDCD2 influences assembly of the ribosome and transcript-specific RNPs independently, we favor the hypothesis that Zfrp8/PDCD2 provides a functional link between the transcripts and ribosomes (Fig 5). For instance, by assisting the formation of competent transcript-specific-RNPs in the nucleus, Zfrp8 may prevent their premature binding to pre-40S and translation. Recent studies suggest that the pre-40S/40S subunit may be able to bind mRNA and initiate translation in the nucleus [52, 60–62]. However, the vast majority of protein synthesis happens in the cytoplasm, and eukaryotic cells utilize check point mechanisms to prevent binding of immature subunits to mRNA to avoid unnecessary nuclear translation [33, 63]. If Zfrp8/PDCD2 is part of such a check point mechanism, lack of Zfrp8 in the nucleus may cause improper binding between pre-40S and transcript-specific RNPs or mRNAs, and therefore disrupt their nuclear export. In addition, in the cytoplasm, Zfrp8/PDCD2 may stimulate the binding between RNPs and 40S subunits, facilitating final ribosome assembly, and thereby, stabilize ribosomal proteins, and ensure translation of select transcripts. This step in the control of gene expression is not well studied. Because Zfrp8 is specifically required in stem and rapidly dividing cells, such as cancer cells, our results further confirm the cell type specificity of RNA processing and ribosomal biogenesis. Much additional work will be necessary to understand how these specific mechanisms are linked to cell fate.

## Materials and Methods

### Fly lines and genetic interactions

The GFP-trap lines ([45, 46], S2 Table) were obtained from the Bloomington Stock Center, and GFP-trap-RpS2 line, P{PTT-un1} ZCL3170 were from L. Cooley, (School of Medicine, *Yale* University, CT, USA). *RpS2*^*P*^ *(sop*^*P*^, [44]) line was obtained from F.A Laski (University of California, CA, USA),*UAS-Venus* was from D. Ish-Horowicz, (University College, London, UK). *RpS2*^*PRW1*^, other mutant alleles, balancer and driver stocks were from Bloomington Stock Center (IN, USA).

The UAS-GFP and RFP constructs for ribosomal proteins (Table 1, Fig 3, [48] were provided by S. Brogna (University of Birmingham, UK) and S. Speese (Oregon Health and Science University OR, USA). The UASp-mCherry-RpS2 construct was created using the pUAS-N-mCherry-BD-attB vector, and transgenic flies were generated by the insertion in attP2 site [64, 65].

Zfrp8 germ line- and soma specific KD in ovaries were done as previously discribed [8]. For *Zfrp8 KD* and somatic *Zfrp8 KD(s)* in ovaries (*nos-GAL4/+; UAS-Zfrp8 RNAi {GL00541}*, *nos-GAL4/UAS-Zfrp8 RNAi {HMJ02095}*, *tj-GAL4/+; UAS-Zfrp8 RNAi* {GD4600}/*tub-GAL80*^*ts*^) flies were raised at 29°C. For most experiments, ovaries dissected from 0-16h females were used. Additionally, for *RpS2* mutants and *tj-GAL4* combinations we analyzed ovaries from 5 and 7 days old females. Appropriate driver alone and *UAS-RNAi* alone controls were used for each experiment. At least 10 females of each genotype were analyzed.

To compare GFP and RFP tagged protein distribution in KD and control tissues, the KD and control flies were grown in parallel under the same conditions, 4–6 pairs of ovaries of each genotype were fixed, mounted and scanned. Each experiment was repeated 3 times.

The *tub-GAL4/ GFP-TRAP-RpS2; UAS-Zfrp8 RNAi* {GD4600}/tub-GAL80^ts^ to obtain tissues with general *Zfrp8* knock-down were raised at 18°C until the second instar larval stage, transferred to 29°C. Brains were dissected from late third instar larvae and analyzed by western blotting.

### Zfrp8/PDCD2 two-hybrid screen and TAP-(Tandem Affinity Purification) experiments

The two-hybrid screen was performed as described in [14]. For bait, full-length Zfrp8 was cloned into the pGBKT7 vector. Expression of Zfrp8 in yeast cells was confirmed by Western blotting using anti-Zfrp8, and tested for auto-activation and toxicity. The pGBKT7-Zfrp8 construct was then mated to a Mate and Plate normalized *Drosophila* universal library (#630485, Clontech, Mountain View, CA). Positive colonies were confirmed on high-stringency plates containing (-Ade/-His/-Leu/-Trp/X-α-Gal/AbA). Retested positives were then sequenced to identify Zfrp8 interactors.

For TAP experiments, full-length human PDCD2 [3] was cloned into pCEMM-NTAP [66]. HEK293 cells were grown in DMEM (Gibco) medium in the presence of 10% fetal calf serum. Cells were transfected with 1 μg of pCEMM-TAP-PDCD2 and pCEMM-NTAP control plasmid using Lipofectamine 200 reagent (Invitrogen) according to manufacturer’s instructions. 5X10^6^ cells were used for TAP.

TAP was done as described in [66–68]. Briefly, transfected cells were washed three times with cold PBS, lysed with lysis buffer (50 mM Tris pH 7.5, 125 mM NaCl, 5% glycerol, 0.2% IGEPAL, 1.5 mM MgCl_2_, 1 mM DTT, 25 mM NaF, 1 mM Na_3_VO_4_, 1 mM EDTA, Complete protease inhibitor, Roche) for 20 min on ice. Extracts were cleared by centrifugation at 13,000x rpm for 20 min and incubated with IgG agarose (Sigma) at 4°C for 2h, washed with lysis buffer and with TEV-protease cleavage buffer (10 mM Tris pH 7.5, 100 mM NaCl, 0.1% IGEPAL, and 0.5 mM EDTA), than eluted by addition of 40 μg TEV protease under vigorous shaking for 1.5 h at 16°C. TEV-protease cleavage products were incubated with Ultralink Immobilized Streptavidin Plus beads (Pierce) at 4°C for 1 h. After 4 washes proteins were eluted in SDS-PAGE-sample buffer at 90°C for 10 min. Eluates were analyzed by western blotting. Zfrp8 TAP one-step purification was essentially done as described in [14, 68]. We used ovaries instead of embryos, IgG agarose binding and TEV-cleavage steps were skipped, and cleared extracts were directly bound to Streptavidin Plus beads (Pierce).

### FISH

Fluorescent in situ hybridizaton (FISH) was done as described in [69, 70] using, HRP-anti-DIG AB (Jackson Laboratories) and Cy3 tyramid conjugate. DIG labeled RNA probes were synthesized using T7 RNA polymerase and DIG-RNA labeling kit (Roche) from gene specific PCR fragments (primers are listed in S3 Table).

### Antibodies and microscopy

Rabbit anti-Zfrp8 antibody [8] were used at 1:1000–1:2000, Rabbit anti-RpS2 (ARP63572, Aviva systems Biology) at 1:1000, rabbit anti-PDCD2 (from P. Sharp, MIT, MA, USA, REF) 1:2000. G*uinea pig anti*-Traffic Jam (*Tj*) (1:5000, from D. Godt, Toronto, Canada); rabbit anti-Bazooka (Baz) (1:500 from A. Wodarz); mouse anti-1B1 antibody (1:20, developed by H. Lipshitz, from the Developmental Studies Hybridoma Bank (Iowa University, Iowa City, IA, USA); and rabbit anti-RanGAP (1:700, from B. Ganetzki), were used to mark follicle cells, spectrosomes, cytoplasm and the nuclear envelope in ovaries. Alexa Fluor-*546* phalloidin (Invitrogen) and secondary antibodies (Jackson Laboratories) were used at 1:300. Hoechst 33258 (1:5000) and Vectashield with DAPI (vector Laboratories) were used to stain DNA. Images were captured using a Leica TSC SP8 or Leica TSC SP5 laser scanning confocal microscopes (objective 63× oil), analyzed with Leica Microsystems software and further processed using Adobe Photoshop.

### Quantitative RT-PCR

RNA was isolated from 20–40 young (0-16h) ovaries using RNeasy Plus Mini kit (Qiagen). RT-reactions were performed using M-MLV reverse transcriptase (Invitrogene) and random primers. Oligo-dT was used in parallel experiments, and similar results for all tested transcripts were obtained (data not shown). Quantitative PCR was performed as described in the manufacturer’s instructions using the SYBR Select Master Mix, Step One Plus Real Time PCR System (Applied Biosystems, Austin, TX), and the relative standard curve method. Primers used for RT-PCR are listed in S3 Table. Transcript levels were normalized to those of *RpL32* and *GAPDH2*. *nos-GAL4/+*, *tj-GAL4/+* and *UAS-Zfrp8* RNAi/+ were used as control ovaries, and all data were normalized to the transcript levels in *w118* ovaries (baseline = 1). At least two biological and two technical replicates were performed for each genotype. Statistical significance (*P*-value) was determined using two-tailed Student’s *t*-test.

## Supporting Information

S1 Fig Genetic interaction between Zfrp8 and RpS2. (A-D) The levels and distribution of Zfrp8 (red) is not affected in *RpS2* mutants (C-D) compared to that in control (A-B). (E) Typical round shape and rare post-mitotic *exclamation shape spectrosomes (arrows) in control germarium*. (F, G) ~40% of spectrosomes (arrows) in *RpS2*^*P*^ GSCs had extended, symmetrical, or dumbbell shapes (compare to E, *arrows)*. (H, J) *RpS2* ^*P*^ phenotype (H) is enhanced by lack of one copy of *Zfrp8* (J), egg chambers become smaller and ultimately the germ line cells are lost (arrow). (I, K, L) Similar phenotypes were observed in *Zfrp8* KD ovaries combined with heterozygous *RpS2* mutations (two *RpS2* alleles used, compare K and L to I). (A-G) ovaries dissected from 1-16h females. (H-L) ovaries from 4–5 days old females (see Materials and methods); DNA blue (DAPI), F-actin red (phalloidin), size bar is 20μm.
(TIF)

S2 Fig Zfrp8 regulates RpS2, but does not affect expression of Venus or RpS18. (A-B) *nos-GAL4* driven expression of Venus (green) reflects the *nos* expression pattern in control ovaries. (C-D) In *Zfpr8* KD ovaries the Venus expression pattern was altered reflecting the phenotype, but the levels of expression were not reduced. (E-H) *nos-GAL4* driven expression of GFP-RpS18 (green) was not reduced in *Zfrp8* KD, but changed concurrently to the phenotype (G-H). (I-L) Levels of mCherry-RpS2 (*nos-GAL4*, *UAS-mCherryRpS2*) were dramatically reduced in *Zfrp8* KD ovaries (compare I-J and K-L DNA blue (DAPI), size bar is 20μm.
(TIF)

S3 Fig Effect of *Zfrp8* on protein expression levels in ovaries. Examples of GFP-trap protein expression in control and *Zfrp8 KD* ovaries. (A-B) Zn72D (green) was seen in the nuclei of germ line cells (arrows) and follicle cells (small nuclei). (C-D) Levels and distribution of Zn72D were unaffected in Zfrp8 KD ovaries. (E-F) At early stages of oogenesis Me31B (green) is highly enriched in oocytes (arrowheads), and present in lower levels in all ovarian cells. In egg chambers after stage 6–7 Me31B is also strongly increased in the nurse cells and oocytes (not-shown). (G-H) *Zfrp8* KD egg chambers do not develop beyond stage 4 and showed defects in oocyte specification (G-H, and [8]). However, in *Zfrp8* KD germaria (G, arrow), Me31B was expressed at similar levels as in control (E). (I-J) CycB (green) is present in GSCs and cystoblasts (arrows) and is also observed in most follicle cells. (K-L) The numbers of GSCs and cystoblasts expressing CycB and the level of the protein was similar in control and *Zfrp*8 KD ovaries. (M-T) The translation factors, EIF4E and EF2 showed similar expression levels in *Zfrp8* KD germaria (O, S, arrows) and control (M, Q), but the levels of both proteins are decreased in *Zfrp8* KD egg chambers (O, S, arrowheads). DNA blue (DAPI), size bar 20μm.
(TIF)

S4 Fig Zfrp8 affects cytoplasmic localization of select transcripts. (A-B) Levels and localization of *TAHRE* transcripts (FISH, red) are low in control ovaries and are significantly increased in the cytoplasm of *Armi*^*1*^*/Armi*^*72*.*1*^ ovaries (C-D arrows show GSCs). In *Zfrp8 KD* ovaries (driven by nos-GAL4), levels of *TAHRE* RNA was increased in both nuclei and cytoplasm of germ line cells (E-F, arrow). Increase in *TAHRE* RNA was also observed in follicle cells, suggesting that Zfrp8 may also have non-cell autonomous effect on *TE* regulation [8]. (G-R) FISH with *Pino* (G-J), *sta* (K-N) and *RpL36* (O-R) probes. Levels of *Pino* RNA are somewhat elevated and showed significant nuclear accumulation (G-J, arrows) in *Zfrp8 KD* ovaries. *sta* transcript levels and localization were not changed in *Zfrp8* KD ovaries (K-N, arrows). *RpL36* transcripts showed increased nuclear accumulation in *Zfrp8* KD ovaries (O-R, arrows). To visualize cellular compartments ovaries were counterstained with anti-RanGAP antibodies, green (A, C, E, O and Q, cytoplasm and nuclear envelope), anti-Baz antibodies, green, (G, I, K and M, cytoplasm and apical-lateral membrane of follicle cells) and DAPI, blue (DNA).
(TIF)

S5 Fig Quantification of mRNA fluorescence* in the nuclei and cytoplasm of stage 4–5 nurse cells. (A) *TAHRE* RNA is increased in both nuclei and cytoplasm of *Armi* and *Zfrp8 KD* ovaries compared to wild type controls. The ratio of nuclear to cytoplasmic levels (n/c) is increased in *Zfrp8* KD. (B) *Pino* and *RpL36* (D) mRNAs also show increased nuclear accumulation and elevated n/c ratio in *Zfrp8 KD* ovaries, while the levels and nuclear accumulation of *sta* mRNA (C) and *RpS2* mRNA (E) remain unchanged. Y axis shows mRNA fluorescence* (fluorescence intensity, see below), error bars represent standard deviation. n/c shows average ratio between nuclear and cytoplasmic mRNA fluorescence, p values were calculated using Student t test and n/c ratios from individual cells. *To allow for accurate measurements of mRNA fluorescence for each probe, crosses, ovary dissection, processing (FISH), and imaging were done in parallel. Images were captured using a Leica TSC SP5 laser scanning confocal microscopes (objective 63× oil) with the same microscope settings, scanner and laser intensity. Using Leica Microsystems software we measured mean fluorescence intensity in an area of 25μm^2^ within the nucleus and in the cytoplasm of the nurse cells from stage 4 and 5 egg chambers. Background fluorescence (measurements taken from ovaries hybridized with the sense probe and non-specific probe) were subtracted. Each bar represent an average of 15 measurements collected from 4–5 ovarioles.
(TIF)

S1 Table Potential Zfrp8 interactors identified in two hybrid screen. (DOCX)

S2 Table GFP-trap lines tested in Zfrp8 KD background. The name of the protein, allele, predicted protein function, protein distribution and changes observed in *Zfrp8* KD (*nos-Gal4; UAS-Zfrp8 RNAi {GL00541})* ovaries compared to control (*nos-GAL4/+*).
(DOCX)

S3 Table List of primers. (DOCX)
