# Supplementary figures and images for: Zfrp8/PDCD2 Interacts with RpS2 Connecting Ribosome Maturation and Gene-Specific Translation
Source: PLoS One. 2016 Jan 25;11(1):e0147631. doi: 10.1371/journal.pone.0147631 (PMC4726551; doi:10.1371/journal.pone.0147631)

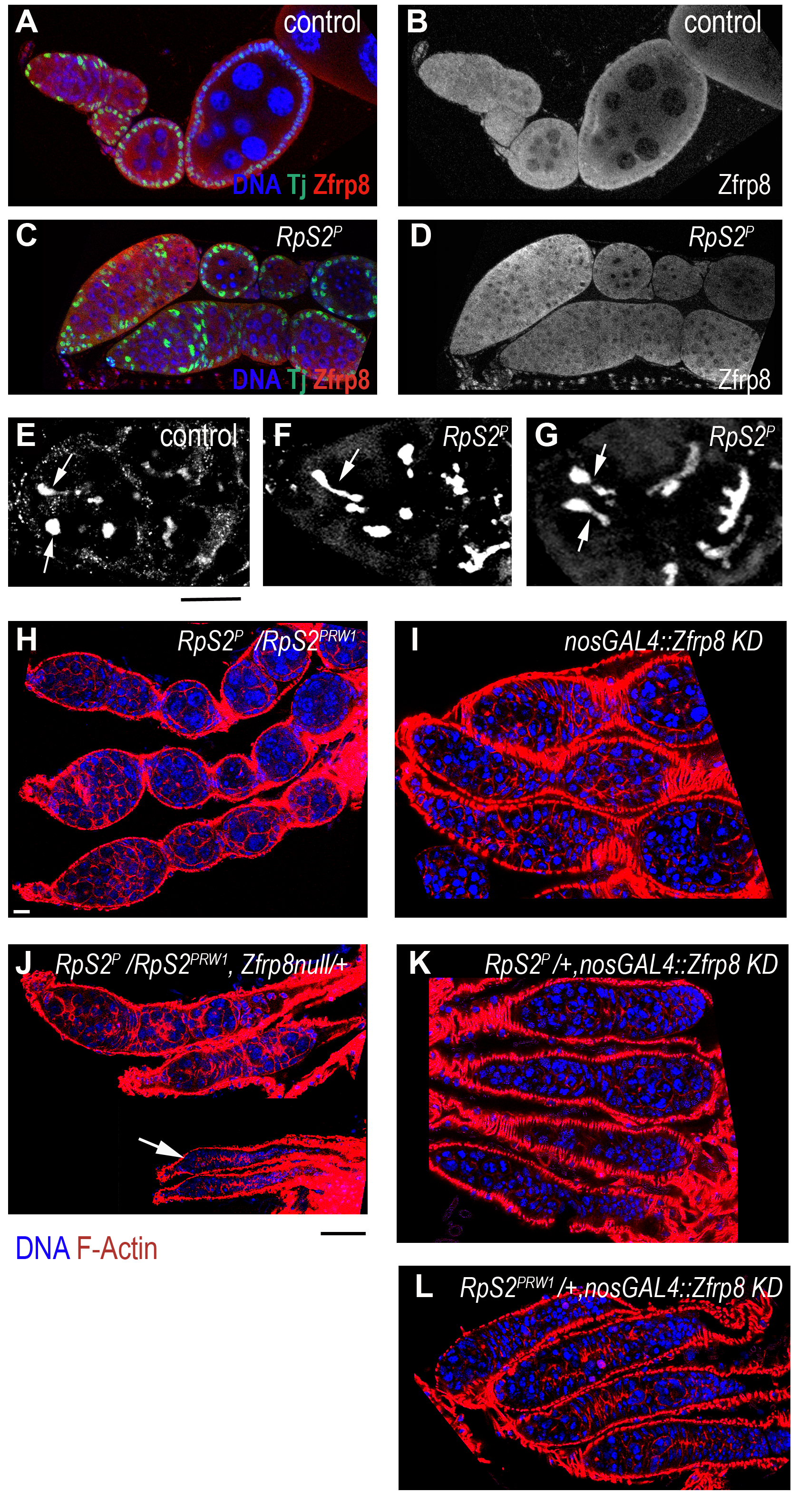

Supplement: S1 Fig — (A-D) The levels and distribution of Zfrp8 (red) is not affected in RpS2 mutants (C-D) compared to that in control (A-B). (E) Typical round shape and rare post-mitotic exclamation shape spectrosomes (arrows) in control germarium. (F, G) ~40% of spectrosomes (arrows) in RpS2P GSCs had extended, symmetrical, or dumbbell shapes (compare to E, arrows). (H, J) RpS2 P phenotype (H) is enhanced by lack of one copy of Zfrp8 (J), egg chambers become smaller and ultimately the germ line cells are lost (arrow). (I, K, L) Similar phenotypes were observed in Zfrp8 KD ovaries combined with heterozygous RpS2 mutations (two RpS2 alleles used, compare K and L to I). (A-G) ovaries dissected from 1-16h females. (H-L) ovaries from 4–5 days old females (see Materials and methods); DNA blue (DAPI), F-actin red (phalloidin), size bar is 20μm. (TIF) [file pone.0147631.s001.tif]

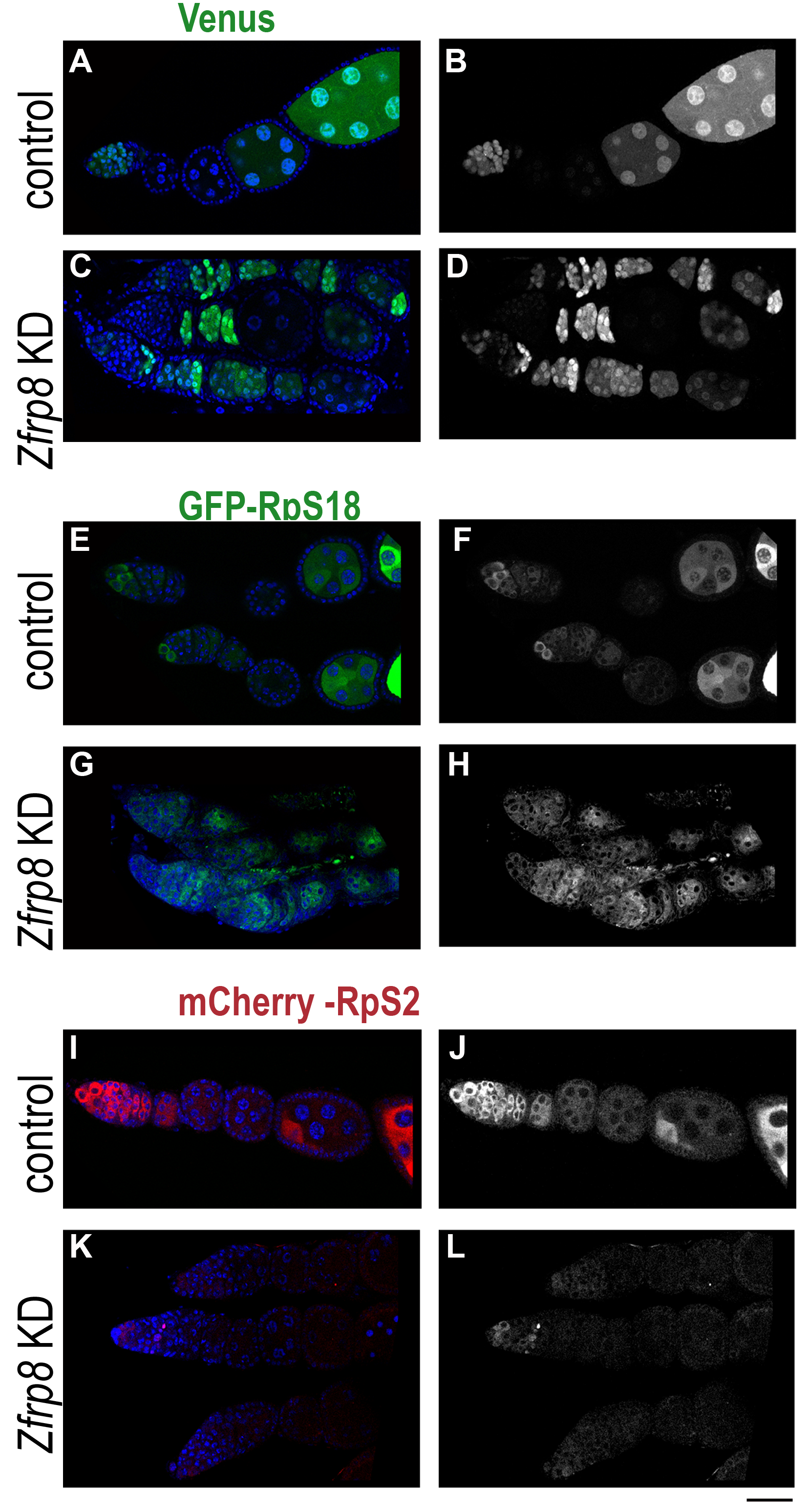

Supplement: S2 Fig — (A-B) nos-GAL4 driven expression of Venus (green) reflects the nos expression pattern in control ovaries. (C-D) In Zfpr8 KD ovaries the Venus expression pattern was altered reflecting the phenotype, but the levels of expression were not reduced. (E-H) nos-GAL4 driven expression of GFP-RpS18 (green) was not reduced in Zfrp8 KD, but changed concurrently to the phenotype (G-H). (I-L) Levels of mCherry-RpS2 (nos-GAL4, UAS-mCherryRpS2) were dramatically reduced in Zfrp8 KD ovaries (compare I-J and K-L DNA blue (DAPI), size bar is 20μm. (TIF) [file pone.0147631.s002.tif]

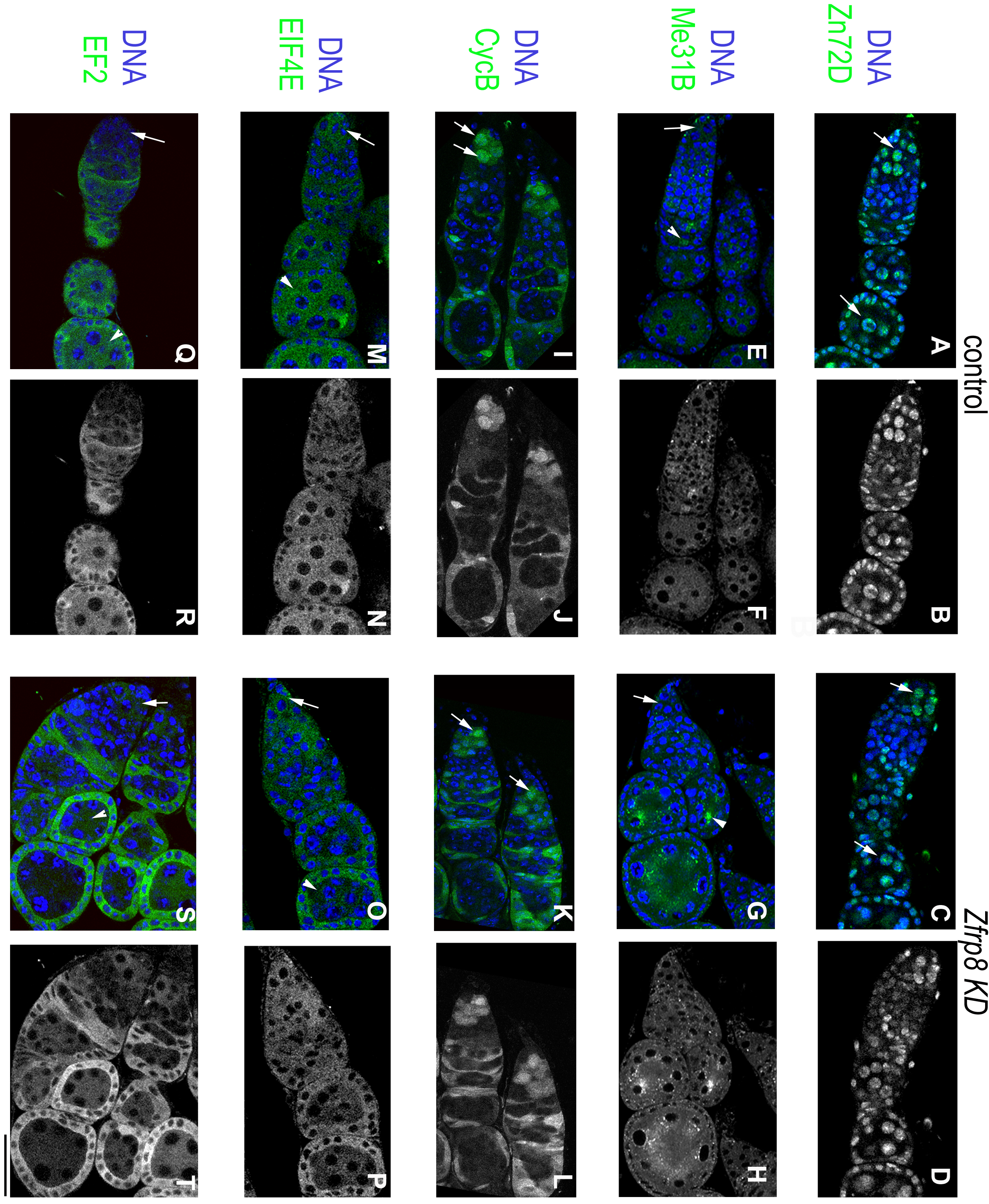

Supplement: S3 Fig — Examples of GFP-trap protein expression in control and Zfrp8 KD ovaries. (A-B) Zn72D (green) was seen in the nuclei of germ line cells (arrows) and follicle cells (small nuclei). (C-D) Levels and distribution of Zn72D were unaffected in Zfrp8 KD ovaries. (E-F) At early stages of oogenesis Me31B (green) is highly enriched in oocytes (arrowheads), and present in lower levels in all ovarian cells. In egg chambers after stage 6–7 Me31B is also strongly increased in the nurse cells and oocytes (not-shown). (G-H) Zfrp8 KD egg chambers do not develop beyond stage 4 and showed defects in oocyte specification (G-H, and [8]). However, in Zfrp8 KD germaria (G, arrow), Me31B was expressed at similar levels as in control (E). (I-J) CycB (green) is present in GSCs and cystoblasts (arrows) and is also observed in most follicle cells. (K-L) The numbers of GSCs and cystoblasts expressing CycB and the level of the protein was similar in control and Zfrp8 KD ovaries. (M-T) The translation factors, EIF4E and EF2 showed similar expression levels in Zfrp8 KD germaria (O, S, arrows) and control (M, Q), but the levels of both proteins are decreased in Zfrp8 KD egg chambers (O, S, arrowheads). DNA blue (DAPI), size bar 20μm. (TIF) [file pone.0147631.s003.tif]

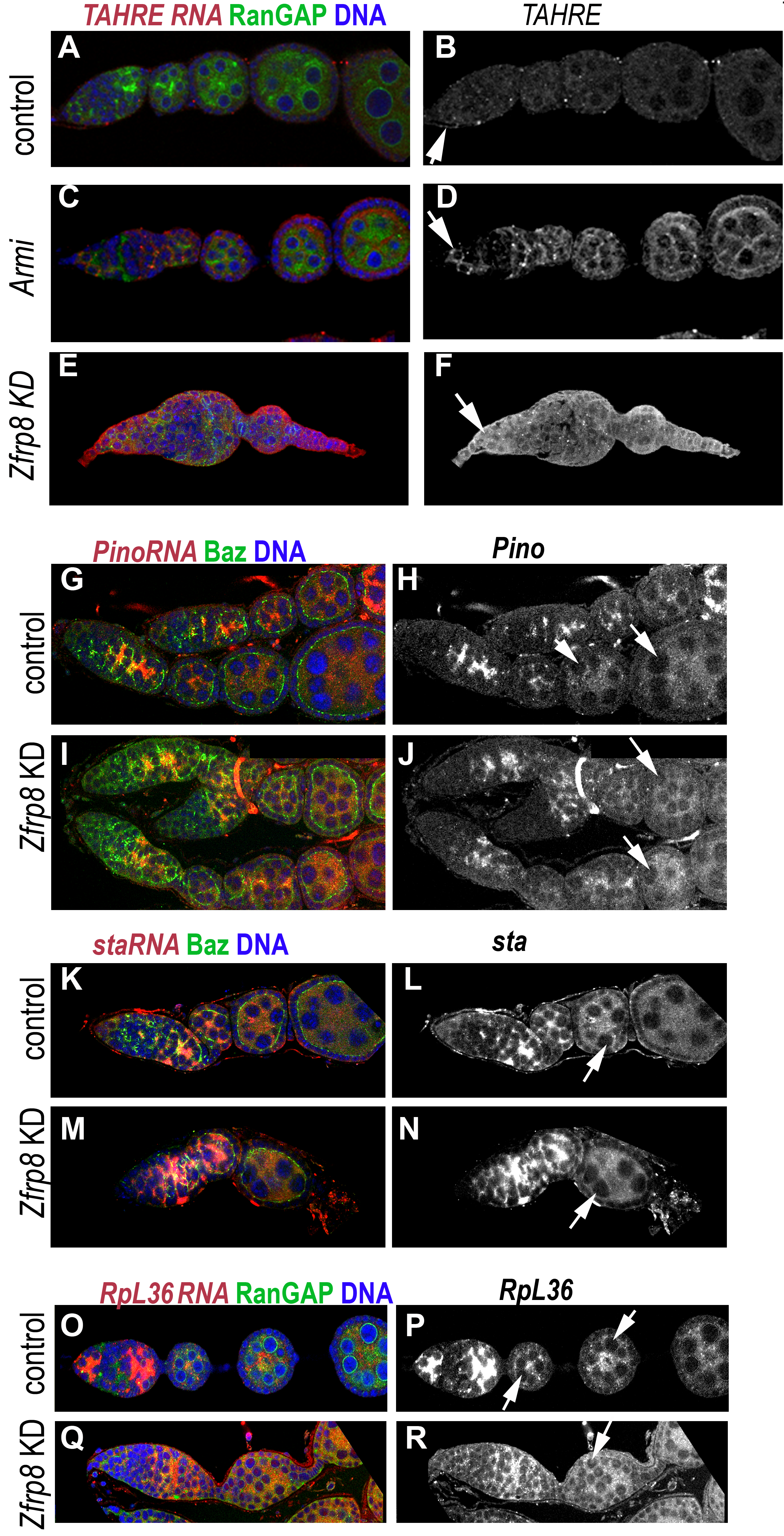

Supplement: S4 Fig — (A-B) Levels and localization of TAHRE transcripts (FISH, red) are low in control ovaries and are significantly increased in the cytoplasm of Armi1/Armi72.1 ovaries (C-D arrows show GSCs). In Zfrp8 KD ovaries (driven by nos-GAL4), levels of TAHRE RNA was increased in both nuclei and cytoplasm of germ line cells (E-F, arrow). Increase in TAHRE RNA was also observed in follicle cells, suggesting that Zfrp8 may also have non-cell autonomous effect on TE regulation [8]. (G-R) FISH with Pino (G-J), sta (K-N) and RpL36 (O-R) probes. Levels of Pino RNA are somewhat elevated and showed significant nuclear accumulation (G-J, arrows) in Zfrp8 KD ovaries. sta transcript levels and localization were not changed in Zfrp8 KD ovaries (K-N, arrows). RpL36 transcripts showed increased nuclear accumulation in Zfrp8 KD ovaries (O-R, arrows). To visualize cellular compartments ovaries were counterstained with anti-RanGAP antibodies, green (A, C, E, O and Q, cytoplasm and nuclear envelope), anti-Baz antibodies, green, (G, I, K and M, cytoplasm and apical-lateral membrane of follicle cells) and DAPI, blue (DNA). (TIF) [file pone.0147631.s004.tif]

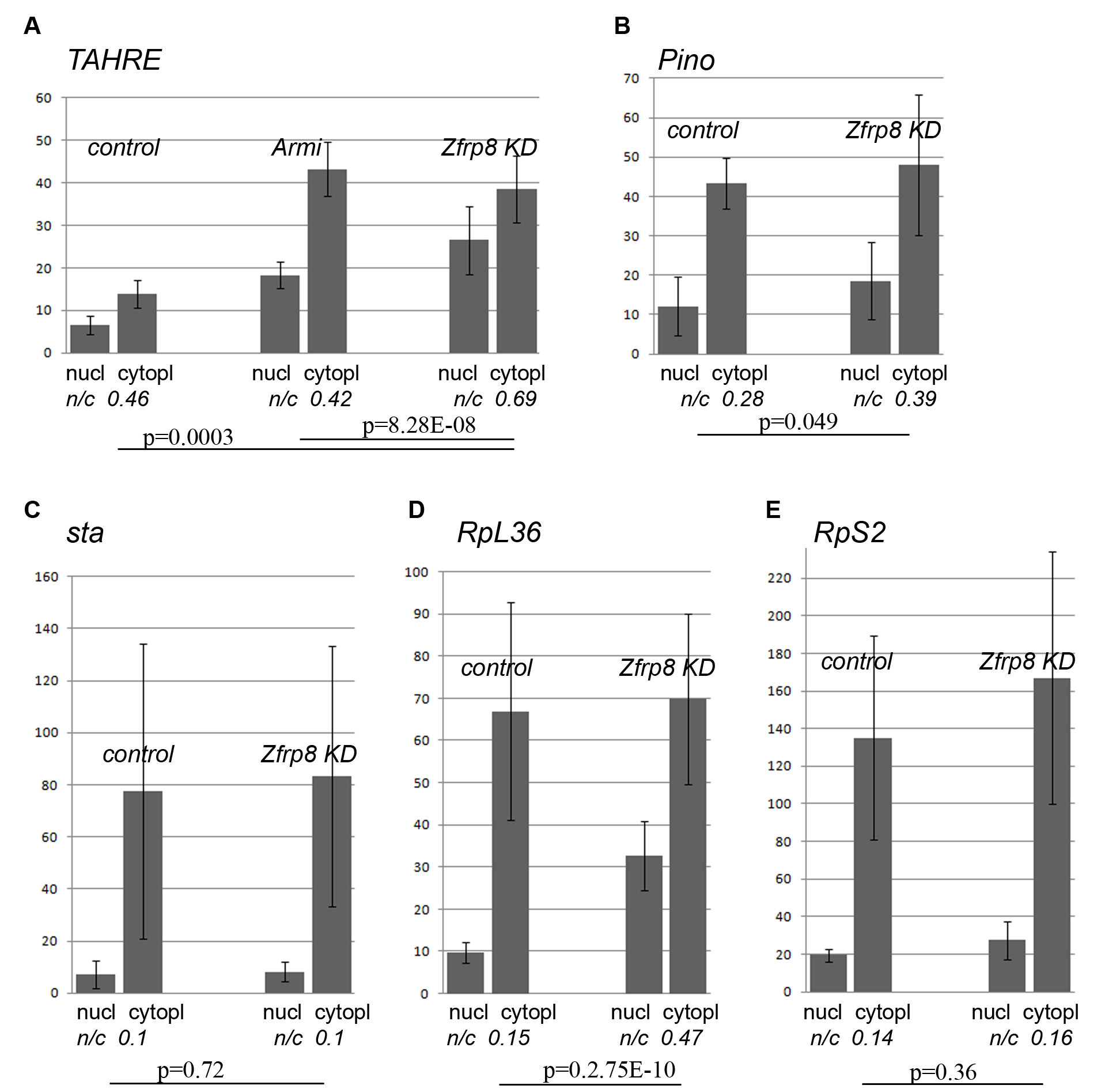

Supplement: S5 Fig — (A) TAHRE RNA is increased in both nuclei and cytoplasm of Armi and Zfrp8 KD ovaries compared to wild type controls. The ratio of nuclear to cytoplasmic levels (n/c) is increased in Zfrp8 KD. (B) Pino and RpL36 (D) mRNAs also show increased nuclear accumulation and elevated n/c ratio in Zfrp8 KD ovaries, while the levels and nuclear accumulation of sta mRNA (C) and RpS2 mRNA (E) remain unchanged. Y axis shows mRNA fluorescence* (fluorescence intensity, see below), error bars represent standard deviation. n/c shows average ratio between nuclear and cytoplasmic mRNA fluorescence, p values were calculated using Student t test and n/c ratios from individual cells. *To allow for accurate measurements of mRNA fluorescence for each probe, crosses, ovary dissection, processing (FISH), and imaging were done in parallel. Images were captured using a Leica TSC SP5 laser scanning confocal microscopes (objective 63× oil) with the same microscope settings, scanner and laser intensity. Using Leica Microsystems software we measured mean fluorescence intensity in an area of 25μm2 within the nucleus and in the cytoplasm of the nurse cells from stage 4 and 5 egg chambers. Background fluorescence (measurements taken from ovaries hybridized with the sense probe and non-specific probe) were subtracted. Each bar represent an average of 15 measurements collected from 4–5 ovarioles. (TIF) [file pone.0147631.s005.tif]
