# Supplementary material for: Zfrp8/PDCD2 Interacts with RpS2 Connecting Ribosome Maturation and Gene-Specific Translation
Source: PLoS One. 2016 Jan 25;11(1):e0147631. doi: 10.1371/journal.pone.0147631 (PMC4726551; doi:10.1371/journal.pone.0147631)
Supplement: S1 Table — (DOCX) [file pone.0147631.s006.docx]

**S1 Table.**

| **Protein name** |  | **Number of independent clones** |
| --- | --- | --- |
| RpS2 | Ribosomal protein S2 | 5 |
| Sds22 | Protein phosphatase 1, regulatory subunit 7 | 3 |
| Rassf | Ras association domain family member 4 | 3 |
| Unc-13 | Protein unc-13 homolog A | 1 |
| RFABG | Apolipoprotein B | 1 |
| Asx | Additional sex combs-like 2 | 2 |
| Dlg5 | Disks large homolog 5 | 2 |
| CG5787 | PIH1 domain-containing protein 1 | 2 |
| CG8366 |  | 1 |
| CG42807 |  | 1 |
| CG31224 | Zinc finger and BTB domain-containing 11 | 1 |
| CG30020 | Zinc finger and BTB domain-containing 41 | 1 |
| CG33278 |  | 1 |

Potential Zfrp8 interactors identified in two hybrid screen
