# Supplementary material for: Zfrp8/PDCD2 Interacts with RpS2 Connecting Ribosome Maturation and Gene-Specific Translation
Source: PLoS One. 2016 Jan 25;11(1):e0147631. doi: 10.1371/journal.pone.0147631 (PMC4726551; doi:10.1371/journal.pone.0147631)
Supplement: S2 Table — The name of the protein, allele, predicted protein function, protein distribution and changes observed in Zfrp8 KD (nos-Gal4; UAS-Zfrp8 RNAi {GL00541}) ovaries compared to control (nos-GAL4/+). (DOCX) [file pone.0147631.s007.docx]

**S2 Table**

| **protein** | **allele** | **function** | **distribution in the ovary** | **dubcellular localization** | **change in *Zfrp8* KD** |
| --- | --- | --- | --- | --- | --- |
| Cyclin B | *P{w[+mC]=PTT-GC}CycB^CC01846^* | Cell cycle | GSC, cystoblasts, follicle cells | Nuclear, cytoplasm | no |
| β-Tubulin | *P{PTT-GC}βTub56D^YC0063^* | Cytoskeleton | Ubiquitous | Cytoplasm | no |
| Rab11 | *P{w[+mC]=PTT-GA}Rab11^CA07717^* | GTPase, endosome recycling | Ubiquitous | Cytoplasm | no |
| Rm62 | *P{PTT-GB}Rm62^YB0077^* | mRNA binding,  RNA helicase | Ubiquitous | Nuclear, cytoplasmic clusters | no |
| Zn72D | *P{PTT-GA}Zn72D^CA07703^* | mRNA binding,  splicing | Ubiquitous | Nuclear | no |
| Mod(Mdg4) | *P{PTT-GA}mod(mdg4)^CA07012^* | Chromatin binding | Ubiquitous, but low in GSCs and cystoblasts | Nuclear | no |
| Jumu | *P{w[+mC]=PTT-GC}jumu^CC00294^* | Transcription factor  Forkheadbox N1 | Ubiquitous | Mostly nuclear. | no |
| Larp | *P{w[+mC]=PTT-GC} larp^ZCL2726^* | RNA binding | Increased in later stage egg chambers | Cytoplasm | no  1* |
| L(1)G0320 | *P{PTT-un1} l(1)G0320^G00024^* | Translocon associated protein | Enriched in GSC, cystoblasts and oocyte (after stage 3) | Cytoplasm, membranes | 1* |
| Me31B | *P{PTT-GB}me31B^CB05282^* | RNA helicase | Ubiqutous,strongly enriched in the oocyte | Cytoplasm | 1* |
| Lost | *P{w[+mC]=PTT-GA}lost^ZCL3169^]* | RNA binding | Increased in egg chambers and oocyte (after stage 5) | Cytoplasm | 1* |
| CG3036 | *P{w[+mC]=PTT-GA}CG3036^CA06801^* | Major facilitator superfamily | Low, enriched in oocyte | Membrane | no |
| EIF-4E | *P{w[+mC]=PTT-GC}eIF-4E^YC0001^* | Eukaryotic translation initiation factor | Ubiquitous, enriched in oocyte | Cytoplasm | 2* |
| EF2 | *P{PTT-GC}EF2^CC01924^* | Eukaryotic translation elongation factor | Ubiquitous, Increased in later stage egg chambers | Cytoplasm | 2* |
| Jupiter | *P{w[+mC]=PTT-GA}Jupiter^G00147^* | Zn finger,  microtubule binding | Ubiquitoous, enriched in GSC cystoblasts, follicle cells | Cytoplasm, nuclear | no |
| Kis | *P{w[+mC]=PTT-GC}kis^CC01466^* | Chromodomain DNA helicase | GSCs and somatic cells | Nuclear | no |
| Pkn1 | *P{PTT-GC}Pkn^CC01654^* | Protein kinase N1  cell motility, cell cycle | Somatic cells | Cytoplasm | no |
| GFP-trap lines tested in Zfrp8 KD background. The name of the protein, allele, predicted protein function, protein distribution and changes observed in *Zfrp8* KD (*nos-Gal4; UAS-Zfrp8 RNAi {GL00541})* ovaries compared to control (*nos-GAL4/+*)  1* no change in germarium and stage 1 oocyte, but the levels may be reduced at later stages due to lack of oocyte specification  2* no change in germarium and stage 1 oocyte, but the levels are reduced in Zfrp8 KD egg chambers arrested in their development. | | | | | |
