# Supplementary material for: Zfrp8/PDCD2 Interacts with RpS2 Connecting Ribosome Maturation and Gene-Specific Translation
Source: PLoS One. 2016 Jan 25;11(1):e0147631. doi: 10.1371/journal.pone.0147631 (PMC4726551; doi:10.1371/journal.pone.0147631)
Supplement: S3 Table — (DOCX) [file pone.0147631.s008.docx]

**S3 Table. List of primers**

| gene/ transcript | forward primer 5' → 3' | reverse primer 5' → 3' |
| --- | --- | --- |
| Templates for probes | | |
| *RpS2* | TAATACGACTCACTATAGGGTAGTCACTATGAGACTTGCGGC | AGGCATCGGCCTGCAGACGAGG |
| *RpS2* | CCGTCCAGAAGCAGACCCGTGC | TAATACGACTCACTATAGGGCCTCGAAAGTGTTCACTTAG |
| *Pino* | TCACGGTCCCCAAATCGCCGACATC | TAATACGACTCACTATAGGGCCTTTGTCTTTTACAAAACATATCTC |
| *Sta* | TCACGCCCGGTGCCTTCACCAAC | TAATACGACTCACTATAGGGCTGTGCTGATGTAGTGCCATCTG |
| *RPL36* | TCCAACTGCCATACTGTTTCCTGTCC | TAATACGACTCACTATAGGGTTTAATTCTCAAGTAACGTCATCG |
| *TAHRE* | TCAACCTAAATCAAAACTACC | TAATACGACTCACTATAGGGGAGGTCATAATTAAAGGGT |
| *TAHRE* | GCCTTACACAACGAGTGGAAA | TAATACGACTCACTATAGGGGTGAGGTGTGCTAAGGTAGACTC |
| qRT-PCR | | |
| *RpS2* | GCAACAAGATCGGCAAGC | CGAGACAATGCCAGTACCA |
| *sta* | CCATTCCGTGCAACAACAAG | GAAGAACAGATCGACGACTACG |
| *RpL36* | AGACTTCGAAGATCAGGAATGTT | CTTGGACACCTTCAGCAACTC |
| *RpL36 nst* | AGACTTCGAAGATCAGGAATGTT | AAGAGTAGAGTCGTCCGTGTAAA |
| *RpL36 nst* | CACGCACAAACGGGAAAGA | GGGCTTCACCTAGCACAAATC |
| *Pino* | TGCGCACATTACCTGTTCTATC | GCTCACGATTCTTTCTGGCTAA |
| *Pino nst* | GCCATTAACGTTTCGCTTCC | GTGCGTCATCTCGTGTAGTT |
| *pre-rRNA* | GTGCGTAATTGTGGCGAGTA | TTTGATTCGACTTCCACTTTCG |
| *18S rRNA* | GTTCGTGGAGTGATTTGTCTGG | CAGGTACGGCTCCACTTACATA |
| *Gapdh2* | CGTTCATGCCACCACCGCTA | CCACGTCCATCACGCCACAA |
| *TAHRE* | GCCTTACACAACGAGTGGAAA | TGAGGTGTGCTAAGGTAGACTC |
| *TAHRE-pol* | ACGACGGCTTGCTATATAAACT | TGTTTCACCGTCGATCCTTAC |

* *TE* sequences from the genome non-canonical.

nst - primers for non-spliced transcript
